# Supplementary material for: A Critical View of the Application of the APEX Software (Aqueous Photochemistry of Environmentally-Occurring Xenobiotics) to Predict Photoreaction Kinetics in Surface Freshwaters
Source: Molecules. 2019 Dec 18;25(1):9. doi: 10.3390/molecules25010009 (PMC7017383; doi:10.3390/molecules25010009)
Supplement: Supplementary file 1 [file molecules-25-00009-s001.zip › 3.2.4_gcc-4.4.0.zip!/3.2.4_gcc-4.4.0/3.2.4_gcc-4.4.0/doc/octave/html/interpreter/Short_002dcircuit-Boolean-Operators.html]

Short-circuit Boolean Operators - Untitled


Previous: Element-by-element Boolean Operators,
Up: Boolean Expressions


---

#### 8.5.2 Short-circuit Boolean Operators

Combined with the implicit conversion to scalar values in `if` and
`while` conditions, Octave's element-by-element boolean operators
are often sufficient for performing most logical operations. However,
it is sometimes desirable to stop evaluating a boolean expression as
soon as the overall truth value can be determined. Octave's
short-circuit boolean operators work this way.

boolean1 `&&` boolean2: The expression boolean1 is evaluated and converted to a scalar using the equivalent of the operation `all (`boolean1`(:))`. If it is false, the result of the overall expression is 0. If it is true, the expression boolean2 is evaluated and converted to a scalar using the equivalent of the operation `all (`boolean1`(:))`. If it is true, the result of the overall expression is 1. Otherwise, the result of the overall expression is 0. **Warning:** there is one exception to the rule of evaluating `all (`boolean1`(:))`, which is when `boolean1` is the empty matrix. The truth value of an empty matrix is always `false` so `[] && true` evaluates to `false` even though `all ([])` is `true`. boolean1 `||` boolean2: The expression boolean1 is evaluated and converted to a scalar using the equivalent of the operation `all (`boolean1`(:))`. If it is true, the result of the overall expression is 1. If it is false, the expression boolean2 is evaluated and converted to a scalar using the equivalent of the operation `all (`boolean1`(:))`. If it is true, the result of the overall expression is 1. Otherwise, the result of the overall expression is 0. **Warning:** the truth value of an empty matrix is always `false`, see the previous list item for details.

The fact that both operands may not be evaluated before determining the
overall truth value of the expression can be important. For example, in
the expression

```
     a && b++
```

the value of the variable b is only incremented if the variable
a is nonzero.

This can be used to write somewhat more concise code. For example, it
is possible write

```
     function f (a, b, c)
       if (nargin > 2 && ischar (c))
         ...
```

instead of having to use two `if` statements to avoid attempting to
evaluate an argument that doesn't exist. For example, without the
short-circuit feature, it would be necessary to write

```
     function f (a, b, c)
       if (nargin > 2)
         if (ischar (c))
           ...
```

Writing

```
     function f (a, b, c)
       if (nargin > 2 & ischar (c))
         ...
```

would result in an error if `f` were called with one or two
arguments because Octave would be forced to try to evaluate both of the
operands for the operator &.
